# Improving Longitudinal Survey Participation Among Internal Medicine Residents: Incorporating Behavioral Economic Techniques and Avoiding Friday or Saturday Invitations

**DOI:** 10.1007/s11606-019-04836-8

**Published:** 2019-02-06

**Authors:** Krisda H. Chaiyachati, Jason Roy, David A. Asch, C. Jessica Dine, Sanjay Desai, Lisa M. Bellini, Judy A. Shea

**Affiliations:** 1 The Department of Medicine, University of Pennsylvania Perelman School of Medicine, 423 Guardian Drive, 13th Floor Blockley Hall, Philadelphia, PA 19104 USA; 2 The Leonard Davis Institute of Health Economics, University of Pennsylvania, Philadelphia, USA; 3 Department of Biostatistics and Epidemiology, Rutgers School of Public Health, Piscataway, NJ USA; 4 Corporal Michael J. Crescenz VA Medical Center, Philadelphia, PA USA; 5 The Department of Medicine, Johns Hopkins University, Baltimore, MD USA

## BACKGROUND

Low participation rates limit the generalizability of surveys. Prior studies show participation is better with financial incentives.^1, 2^ Beyond financial incentives, insights from behavioral economics reveal that psychological concepts, like regret aversion,^3^ can increase participation. For example, subsequent medication adherence improves when patients are entered into a “regret lottery”—they can win prizes if they adhere or are told how much they could have won, had they adhered.^4^ Other studies of survey participation have compared response rates for some, but not all days of the week.^5^ Using a longitudinal survey of internal medicine residents, we examined the effect of (a) whether the prize and regret message components of a regret lottery increased subsequent participation and (b) whether response rates varied based on the day of the week survey invitations were sent.

## METHODS

Residents from 63 internal medicine programs were invited to complete web-based surveys for the Individualized Comparative Effectiveness of Models Optimizing Patient Safety and Resident Education (iCOMPARE) trial, a cluster-randomized trial designed to test alternative resident duty hour rules.^6^ Residents received one survey, with 24 h to respond, every 2 weeks for 16 consecutive cycles from September 2015 through April 2016. Before each cycle, one first-year and one junior or senior resident at each program was randomly pre-selected to win a prize (either a $25 or $100 Amazon gift card) and randomly assigned a day of the week for survey invitation. At the end of each cycle, pre-selected residents who responded were emailed the prize. Pre-selected residents who had not responded received a “regret message” indicating what they would have won, had they responded. This procedure was repeated each cycle.

We fit a logistic regression model with survey completion (“responding”) as the outcome. The model included two binary variables for the prior cycle’s (a) lottery selection and (b) survey response, and an interaction term between the two. We used generalized estimating equations to account for clustering by resident, adjusting for resident year, trial arm, training program, cycle number, and cumulative prior response rate. In a secondary analysis, we distinguished the effects of winning or regretting by dollar amounts ($25 or $100). The University of Pennsylvania institutional review board approved the protocol. Analyses were conducted using Stata v14.0 (StataCorp LLP).

## RESULTS

In total, 97,142 survey invitations went to 6228 residents, with a 40.0% overall participation rate (Table 1). Non-responders who received a regret message improved their subsequent response probability (OR 1.37, 95% CI 1.12 to 1.68; *P* < 0.001), as did responders receiving a prize (OR 1.44, 95% CI 1.09 to 1.90; *P* = 0.003) (Table 2). Each represents approximately a 4% increase in participation. No differences were found when comparing $25 versus $100 for either regret notices or receiving a prize (*P* = 0.08 and *P* = 0.83, respectively), although power for these tests was low. No differences were observed comparing the effect of regret messages with prizes (*P* = 0.77). The probability of response was lower when survey invitations were sent on Fridays (OR 0.72, 95% CI 0.65 to 0.79; *P* < 0.0001) and Saturdays (OR 0.84, 95% CI 0.76 to 0.92; *P* < 0.0001), corresponding to responses required by Saturday and Sunday, respectively.

**Table 1:** Characteristics of Eligible Residents

Characteristics	Value
Eligible residents^a^, no.	6228
Survey invitations emailed, no.	97,142
Overall response rate, %	40.0
Training level, no. (%)	
PGY-1	2528 (41)
PGY-2	1896 (30)
PGY-3	1804 (29)
Study arm, no. (%)	
Standard	3211 (52)
Flexible	3017 (48)
Lottery selected, by $ amount, no. (%)	
$25	1406 (23)
$100	464 (7.5)

*PGY*, post-graduate year; United States dollar

^a^Residents were eligible if they were emailed an invitation to complete a survey during the study period. Residents were emailed an invitation if they were on a list of emails provided by each training program. Residents were excluded if they were dropped from the email list for any of the 16 survey cycles if these individuals indicated they were included erroneously because of an incorrect email or they were no longer a trainee at their respective training program. "Study arm" refer to the two trial arms within the Individualized Comparative Effectiveness of Models Optimizing Patient Safety and Resident Education (iCOMPARE) where training programs and all their residents were randomized to the 2011 Accreditation Council for Graduate Medical Education shift-length policies (“standard”) or were not required to adhere to shift-length limits (“flexible”)

**Table 2:** Probability of Responding in the Subsequent Cycle After Having Received a Regret Message or Prize, and the Day of Survey Invitation

Categories	Adjusted OR of Responding^e^ (95% Confidence Interval)	P-value
Effect of a regret message^a^ (+Selected/-Responded)		
Regret message for any $ amount^b^	1.37 (1.12 to 1.68)	<0.001
Regret message for $25^b^	1.26 (1.02 to 1.55)	0.03
Regret message for $100^b^	1.77 (1.28 to 2.44)	0.001
Comparison of regret for $25 vs. $100	1.41 (0.96 to 2.07)	0.08
Effect of receiving a prize (+Selected/+Responded)		
Received prize for any $ amount^c^	1.44 (1.09 to 1.90)	0.003
Received prize for $25^c^	1.46 (1.10 to 1.94)	0.01
Received prize for $100^c^	1.38 (0.87 to 2.18)	0.17
Comparison of receiving $25 vs. $100	1.06 (0.62 to 1.82)	0.83
Regret messaged vs. received prize (any $ amount)	1.05 (0.77 to 1.42)	0.77
Day of the week invitations were sent^d^		
Tuesday	0.98 (0.89 to 1.08)	>0.99
Wednesday	0.92 (0.83 to 1.02)	0.19
Thursday	0.93 (0.84 to 1.02)	0.26
Friday	0.72 (0.65 to 0.79)	<0.0001
Saturday	0.84 (0.76 to 0.92)	<0.0001
Sunday	0.98 (0.89 to 1.08)	>0.99

## DISCUSSION

Understanding the influence of behavioral economic techniques on survey response rates is an important advancement in survey science. Our findings suggest that regret messages were as influential as receiving prizes for increasing subsequent survey participation among residents. Additionally, survey responses expected on weekends had fewer responses. Our study has several limitations. First, this is a study of internal medicine residents and may not be generalizable to other populations. Second, this is a study of participation in repeated surveys. The effects of achieved or anticipated prizes or regret may differ when a single survey is administered. Third, the influence of regret messages and days of the week may have been different had we administered paper-based or mail-in surveys. Additional evaluations are needed to validate our findings in these other settings. However, at minimum, these simple techniques are likely to improve survey participation in a longitudinal survey of internal medicine residents. If behavioral economic techniques and targeting the ideal days of the week can increase participation in other survey designs, their low-cost approach (if not free) could be an important, cost-effective mechanism for improving the generalizability of survey-based findings.

## References

[1] Schweitzer M, Asch DA (1995). Timing payments to subjects of mail surveys: cost-effectiveness and bias. J Clin Epidemiol..

[2] Asch DA, Christakis NA, Ubel PA (1998). Conducting physician mail surveys on a limited budget. A randomized trial comparing $2 bill versus $5 bill incentives. Med Care..

[3] Kahneman D, Tversky A (1979). Prospect theory: an analysis of decision under risk. Econometrica..

[4] Volpp KG, Loewenstein G, Troxel AB (2008). A test of financial incentives to improve warfarin adherence. BMC Health Serv Res..

[5] Pit SW, Vo T, Pyakurel S (2014). The effectiveness of recruitment strategies on general practitioner’s survey response rates - a systematic review. BMC Med Res Methodol..

[6] Desai SV, Asch DA, Bellini LM (2018). Education outcomes in a duty-hour flexibility trial in internal medicine. N Engl J Med..

